# Chromosome-Level Assembly and Annotation of the Pearly Heath *Coenonympha arcania* Butterfly Genome

**DOI:** 10.1093/gbe/evae055

**Published:** 2024-03-16

**Authors:** Fabrice Legeai, Sandra Romain, Thibaut Capblancq, Paul Doniol-Valcroze, Mathieu Joron, Claire Lemaitre, Laurence Després

**Affiliations:** Inria, CNRS, IRISA, University of Rennes, 35000 Rennes, France; IGEPP, INRAE, Institut Agro, University of Rennes, 35653 Le Rheu, France; Inria, CNRS, IRISA, University of Rennes, 35000 Rennes, France; LECA, CNRS, Université Grenoble-Alpes, Université Savoie Mont Blanc, Grenoble, France; CEFE, CNRS, EPHE, IRD, Université de Montpellier, Montpellier, France; CEFE, CNRS, EPHE, IRD, Université de Montpellier, Montpellier, France; Inria, CNRS, IRISA, University of Rennes, 35000 Rennes, France; LECA, CNRS, Université Grenoble-Alpes, Université Savoie Mont Blanc, Grenoble, France

**Keywords:** chromosome-level assembly, *Coenonympha arcania*, butterfly, annotation, Satyrinae

## Abstract

We present the first chromosome-level genome assembly and annotation of the pearly heath *Coenonympha arcania*, generated with a PacBio HiFi sequencing approach and complemented with Hi-C data. We additionally compare synteny, gene, and repeat content between *C. arcania* and other Lepidopteran genomes. This reference genome will enable future population genomics studies with *Coenonympha* butterflies, a species-rich genus that encompasses some of the most highly endangered butterfly taxa in Europe.

**Significance**

A high-quality reference genome is a prerequisite for modern population genetics and conservation genomics projects. *Coenonympha arcania* is part of a species-rich genus, including multiple taxa of high conservation concern and at least 2 intensively studied hybrid species. The newly proposed high-quality and chromosome-level assembly will be a key resource for upscaling the study of the genus to whole-genome data.

## Introduction

Butterflies have been intensively studied for their ecology, biogeography, and evolutionary history and are sensitive indicators of environmental changes, such as habitat fragmentation and climate change (Kühn et al. 2005). The genus *Coenonympha* (Nymphalidae, Satyrinae) is a species-rich group comprising more than 30 species mostly distributed in Eurasia (Kodandaramaiah and Wahlberg 2009), which diverged early in the radiation of Satyrinae and lacks any close relative genus (Kodandaramaiah et al. 2010; Wiemers et al. 2020). The phylogeny of the genus is poorly resolved with controversial conclusions depending on the molecular marker used (Peña et al. 2006; Kodandaramaiah and Wahlberg 2009). The different taxa are easily distinguished by their hindwing patterns (eyespot and color patterns) and are found in contrasted habitats. Some studies report occasional hybridization between species with overlapping distribution ranges and even the formation of stable hybrid species (Capblancq et al. 2015, 2019, 2020). While most *Coenonympha* species can be relatively abundant locally, some have strict habitat preferences and are at high risk of extinction. Three species *C. hero*, *C. tullia*, and *C. oedippus* are critically endangered in Europe and benefit from a high protection status (Settele et al. 2008). Yet, in *Coenonympha*, a basal genus in the Satyrinae sub-family, the lack of reference genome has limited population genetics studies to de novo reconstructed ddRAD loci (Capblancq et al. 2015, 2019, 2020; Després et al. 2019; Sherpa et al. 2022; Kebaïli et al. 2023).

Here, we present a chromosome-level genome assembly and annotation of a representative of the *Coenonympha* genus, the pearly heath *C. arcania* (Linnaeus 1761) (Lepidoptera, Nymphalidae, Satyrinae). It is a relatively common and locally abundant species widely distributed in semi-open dry grasslands in Europe from the northern Mediterranean to south-central Scandinavia and in the east from Turkey to the Urals (Besold et al. 2008). Its genome was generated with a long-read PacBio HiFi sequencing approach, complemented with Hi-C scaffolding. Additional analyses were performed on gene and repeat contents in comparison with 2 other high-quality Satyrinae genomes (*Maniola jurtina* and *Pararge aegeria*) available from the Darwin Tree of Life project (Lohse et al. 2021a, Lohse and Weir 2021b). The synteny of these species genomes was also investigated, additionally including a recently published genome assembly of another *Coenonympha* species: the chestnut heath *C. glycerion* (ENA project PRJEB71111).

## Results and Discussion

### Genome Sequence Statistics

**Fig. 1.**
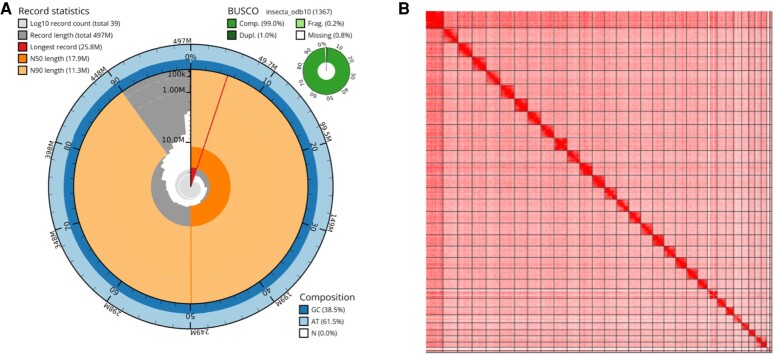
The overview of the *C. arcania* genome assembly. a) Genome assembly statistics shown as a SnailPlot from BlobToolKit, with contiguity (top-left and main circular plot), BUSCO (top right), and GC content (bottom right) metrics. b) The heatmap of chromosomal contact (Hi-C) data. Scaffolds are ordered by decreasing size from left to right, and vertical and horizontal dark lines delineate their boundaries. The color intensity of each pixel represents the frequency of interactions between genomic loci.

HiFi long-read sequencing (PacBio Sequel II) yielded 1.4 million reads (197 Gb, N50 = 14 kb) for the pearly heath butterfly *C. arcania* butterfly, for an estimated genome coverage of 44× (for details, see supplementary table S1, Supplementary Material online). These long reads were assembled into 110 contigs with Hifiasm (Cheng et al. 2021). Next, haplotypic duplications were removed with purge_dups v1.2.5 (Guan et al. 2020), using the HiFi reads and a complementary Chromium 10× library from a *C. arcania* male for the assessment of local coverage, leading to 47 contigs (N50 = 16.5 Mb). Contigs were scaffolded with 141 million Omni-C reads using YaHS v1.2a (Zhou et al. 2023), giving an assembly of 497 Mb in 35 scaffolds (N50 = 18.8 Mb). After visual control of the Hi-C contact map, 3 scaffolds were split resulting in a final assembly of 39 scaffolds including 32 scaffolds larger than 3 Mb and considered to be of chromosome size. Of these 32 scaffolds, we identified the largest scaffold as the Z chromosome, the 2 smallest scaffolds as putatively part of the W chromosome and 29 large autosome-like scaffolds, close to the 28 autosome pairs observed in karyotypes of *C. arcania* (de Lesse 1960). This final assembly has an N50 of 17.9 Mb and BUSCO score of 99.0%, with 98.0% of genes being single copy and complete (Fig. 1a; supplementary fig. S1b and table S3, Supplementary Material online). STAR v2.10.7b (Dobin et al. 2013) reports that 70.0% of the raw RNASeq reads uniquely mapped the genome, while 7.5% mapped at multiple locations (supplementary table S1, Supplementary Material online). When raw reads were trimmed on quality with the nfcore-rnaseq workflow (Ewels et al. 2020), a better mapping rate of 86.59% was achieved.

### Gene Model Predictions and Annotation

Protein-coding genes were annotated using 2 approaches: Braker v3.0.3 (Brůna et al. 2021) complemented by GUSHR (https://github.com/Gaius-Augustus/GUSHR) based on GeMoMa (Keilwagen et al. 2018) and Helixer v0.3.0 (Holst et al. 2023). While the first uses a de novo approach extended with evidence from protein similarities or RNASeq alignments, Helixer implements a deep learning method based only on proteome characteristics from various invertebrate genomes. The number of missing BUSCO genes was slightly higher in the Braker3 annotation, but both methods gave globally good quality results with a similar number of around 21,500 protein-coding genes and retrieved genes of interest, such as wing color pattern genes and chemosensory genes. In order to determine the number of proteins only reported by 1 of 2 methods (i.e. specific to 1 method), we ran Orthofinder (Emms and Kelly 2019) on the 2 annotations, including only the longest isoform for each gene. Around 30% of the proteins were specific to 1 method. Both programs predict untranslated regions (UTR) exons at short distance of the stop codon, in line with other detailed studies from several *Drosophila* species (Sanfilippo et al. 2017; Wang et al. 2019), but UTRs predicted by Braker3 (with RNASeq evidence) are longer and more consistent with the sizes observed in other Nymphalidae (supplementary table S3 and fig. S3, Supplementary Material online, Lo Giudice et al. 2023).

We compared the gene repertoire predicted by Helixer with other Lepidopteran species, namely 2 other Satyrinae species (*M. jurtina* and *P. aegeria*), 4 other Nymphalidae and *Bombyx mori*, using Orthofinder v2.5.5 (Emms and Kelly 2019). Among the 42,461 identified orthogroups, 12,769 include a *C. arcania* protein, and 3,802 genes (17.77%) are specific to *C. arcania* (supplementary table S5 and fig. S4, Supplementary Material online).

### Repeat Content

Based on transposable elements from the Insecta library of RepeatMasker (Smit et al. 2013-2015), an overall similar content in repetitive elements was found in all 3 Satyrinae genomes, respectively 12.27%, 14.87%, and 16.78% for *C. arcania*, *M. jurtina*, and *P. aegeria*. However, the genome of *C. arcania* contains more DNA transposons and LTR elements (supplementary fig. S5a and b, Supplementary Material online), and the de novo approach of RepeatModeler (Flynn et al. 2020) identified a larger proportion of interspersed repeats, namely 43.01%, 37.34%, and 39.24% for *C. arcania*, *M. jurtina*, and *P. aegeria*, respectively, yet around half of these remain unclassified.

### Comparative Analysis

**Fig. 2.**
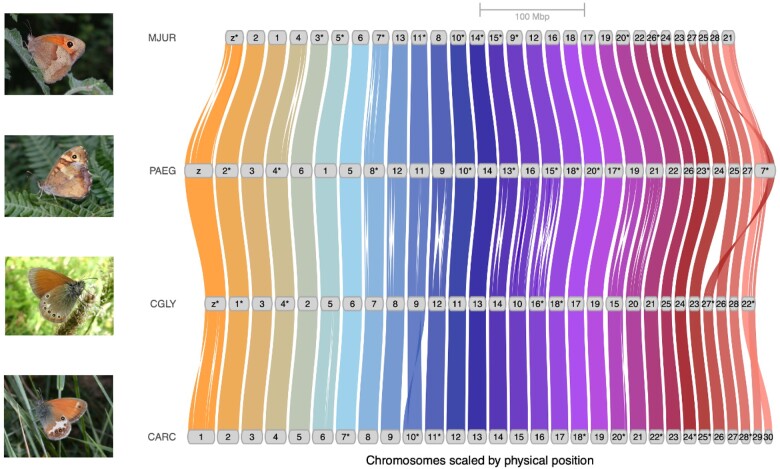
Comparative genomics between *C. arcania* and 3 other Satyrinae genomes. Synteny blocks were obtained by comparing orthologous gene orders on *C. arcania* (CARC, bottom line) with *C. glycerion* (CGLY), *P. aegeria* (PAEG), and *M. jurtina* (MJUR). The stars (*) indicate chromosomal sequences that were reversed to ease the visualization. Photos: Philippe Mothiron (CARC, PAEG, and MJUR) and Jean-Pierre Arnaud (CGLY).

To assess genome synteny among Satyrinae genomes, we compared the *C. arcania* genome against *C. glycerion* (ENA project PRJEB71111), *M. jurtina* (Lohse et al. 2021a), and *P. aegeria* (Lohse and Weir 2021b). Orthologous gene order analyses with GENESPACE (Lovell et al. 2022) revealed a high degree of chromosomal synteny between these genomes, suggesting very few large-scale chromosomal rearrangements between these 4 Satyrinae taxa (Fig. 2). This strong level of synteny was also confirmed by whole genome alignments (supplementary fig. S5c, Supplementary Material online).

This comparative analysis further supports our hypothesis that scaffold 29 and scaffold 30 were misassembled and belong to a single chromosome in the *C. arcania* genome. We expect only 28 autosomes from the karyotype of *C. arcania* (de Lesse 1960), and these 2 scaffolds are the most likely candidates to be fused together, as they appear linked more strongly than any other autosome pair on the Hi-C contact map (Fig. 1b) and show high synteny with a unique chromosome in *C. glycerion, M. jurtina*, and *P. aegeria* (Fig. 2). Unfortunately, we were not able to confirm this fusion with the long reads and therefore kept the 2 scaffolds separated.

### Chromosomes Z and W

To identify sex chromosomes, the coverage of the raw HiFi reads of the female individual (ZW) used for the assembly was compared with Chromium-10× reads obtained from a male individual (ZZ; supplementary table S4, Supplementary Material online). The scaffold 1 shows approximately half the coverage of the other scaffolds in the female only, suggesting it is the chromosome Z. This is corroborated by the strong synteny between scaffold 1 and the Z chromosomes of *C. glycerion, M. jurtina*, and *P. aegeria* (Fig. 2). Unfortunately, we were not able to identify the W chromosome with certainty by comparing the chromosomal median coverage of HiFi reads, probably because of its high repeat content. However, the Chromium-10× reads obtained from a male yield zero median coverage on scaffolds 31 and 32 supporting the hypothesis that these 2 small scaffolds (total size = 6.9 Mb) are part of the sex chromosome W. Furthermore, these 2 scaffolds exhibit a lower rate of polymorphism within mapped Hifi reads compared with autosomal scaffolds, as expected for sexual chromosomes in females and as observed for the Z chromosome (scaffold_1; see supplementary table S4, Supplementary Material online).

## Materials and Methods

### DNA Extraction and Sequencing

High molecular weight DNA was extracted using Gentra Puregene kit from Qiagen, according to the manufacturer's instructions. For the PacBio long-read sequencing performed at the GenoToul Platform (Toulouse, France), DNA was extracted from a freshly emerged *C. arcania* female (BST1) obtained in September 2021 from an egg of a *C. arcania* female caught in June 2021 at La Bastille (Grenoble, France; supplementary fig. S1a, Supplementary Material online). For the 10× genomics sequencing performed at the MGX platform (Montpellier, France), DNA was extracted from a wild caught male *C. arcania* (ARC2) from Saint Michel de Chaillol, France. For the Hi-C library preparation, DNA was extracted from *C. arcania* larvae reared from the eggs of wild caught females from La Mure (France). Final DNA purity and concentrations were measured by spectrometry using Nanodrop and fluorometry using Qubit (ThermoFisher).

### RNA Isolation and Sequencing

Tissues from 1 lab-reared *C. arcania* adult female and 1 *C. arcania* larva were separately extracted with Trizol. mRNA purification, library preparation, and subsequent short-read sequencing (Illumina Novaseq) were performed by NOVOGENE (Cambridge, UK).

### Assembly Quality Assessment

The absence of contamination in the assembly was confirmed by looking at scaffolds with unexpected coverage, GC percent, or similarities (distinguishing putative contaminants), using Blobtoolkit v0.4.7 (Challis et al. 2020) with the HiFi sequences coverage (HiFi reads mapped with minimap2 (Li 2018) with the -ax map-pb option) or the similarities with NCBI NT database (2023-08-21 version) obtained by blastn v2.12.0 (Camacho et al. 2008), with the options -max_target_seqs 10, -max_hsps 1, and -evalue 1e-25. The level of completeness of the assembly was estimated using BUSCO v5.2.2 (Simão et al. 2015) scores against insecta_odb10 and lepidoptera_odb10 gene catalogs. We checked the level of haplotypic duplications caused by high heterozygosity level by comparing kmer abundances in the raw HiFi read set versus in the assembly using DSK v2.3.3 (Rizk et al. 2013) to count 31-mers and display the abundance profiles in a kmer-comparison plot (supplementary fig. S2, Supplementary Material online).

### Identification of Sex Chromosomes

Male (ZZ) read coverage was determined using Chromium 10× reads aligned with bwa mem v 0.7.17 (Li 2013) with default parameters. Alignments with a quality score above 20 and proper pairing were retained using samtools view v1.15 (Danecek et al. 2021) with parameters -q20 -f 0×2. Coverage in 1,000-bp regions was calculated from the resulting bam file using mosdepth v0.3.4 (Pedersen and Quinlan 2018) with options (-b 1000 -n -m). A VCF file was generated from the bam of HiFi reads alignments with bcftools v1.16 (Danecek et al. 2021) mpileup using parameters -Ou, followed by bcftools call -mv -Ob. Variants were selected if covered by at least 5 reads and called heterozygous when the alternative allele depth of coverage ranged from 20% to 80%.

### Annotation of Protein-Coding Genes

Braker v3.0.3 (Brůna et al. 2021) was used with the following inputs: (i) unfiltered bams of the alignments of the raw reads of 2 RNAseq libraries obtained with STAR v2.7b (Dobin et al. 2013) using the options –outFilterMultimapNmax 5 –outFilterMismatchNmax 3 –alignIntronMin 10 –alignIntronMax 50000 –alignMatesGapMax 50000 and (ii) the fasta files of 16 butterflies proteomes downloaded from Lepbase release 4 (http://download.lepbase.org/v4/sequence/, Challi et al. 2016; supplementary table S2, Supplementary Material online) complemented with the proteome of *M. jurtina* (Lohse et al. 2021a). Finally, the GTF file of Braker was supplemented with the UTRs predicted with GUSHR (https://github.com/Gaius-Augustus/GUSHR) based on GeMoMa (Keilwagen et al. 2018) and the bam file from STAR.

In parallel, another set of protein coding genes was annotated using Helixer v0.3.0 (Holst et al. 2023) with the option “–lineage invertebrate,” which corresponds to the matrix invertebrate_v0.3_m_0100, which includes 65 training invertebrate genomes (including *Bicyclus anynana*, *B. mori*, *Papilio machaon*, *Pieris rapae*, and *Plutella xylostella*) and 136 genomes for validation.

Assignation of RNAseq fragments to genes was conducted from bam files after removing potential PCR duplicates with the nfcore rnaseq pipeline v3.10.1 (Ewels et al. 2020) with default parameters (i.e. using the STAR mapper), followed by FeatureCounts from Subread v2.0.1 (Liao et al. 2014). Eighteen wing color pattern genes were extracted from the *Danaus plexippus* V4 genome annotation (Zhan et al. 2011), and 356 chemosensory genes (43 CSP, 40 OBP, 36 IR, 167 GR, and 70 OR) were gathered from the *Ithomia salapia* genome annotation (Gauthier et al. 2023). All were aligned with BLASTP v2.12.0 (Camacho et al. 2008) to the Braker3 and Helixer protein sets with a maximal e-value of 1e−20. *Coenonympha arcania* proteins with 60% identity with a match covering more than 80% of the subject were considered as complete.

### Synteny

The identification of synteny blocks between the genomes of *C. arcania,* 2 other Satyrinii, *M. jurtina* and *P. aegeria* (accessions GCA_905333055.1 and GCA_905163445.1, respectively), and *C. glycerion* (GCA_963855885.1) was performed at the gene level and by aligning whole genome sequences. Because the annotation of the *C. glycerion* genome was not available, we first annotated it using Helixer v0.3.0 (Holst et al. 2023). At the gene level, GENESPACE (Lovell et al. 2022) was used based on the orthogroups calculated by Orthofinder (Fig. 2). Pairwise whole genome alignments were performed with SibeliaZ v1.2.5 (Minkin and Medvedev 2020) with the options –n and -a 16, and the obtained local alignments were clustered in large synteny blocks using maf2synteny v1.2 (Kolmogorov et al. 2018), with a final minimal block size of 500 bp (option -b). Each of the 500+ bp synteny blocks between *C. arcania* and *M. jurtina* was plotted as a Circos link and colored after the *M. jurtina* chromosome of the block. The order of the *C. arcania* chromosomes was rearranged to maximize the visual symmetry of the synteny blocks between the 2 species (supplementary fig. S5, Supplementary Material online).

### Annotation of Transposable Elements

The genomes of *C. arcania, M. jurtina* and *P. aegeria* were compared with the insect library of RepeatMasker v4.1.5 (Smit et al. 2013-2015) with the options “-species insecta -xsmall –gff.” Transposable elements were also identified and classified using a de novo strategy using RepeatModeler v2.0.4 (Flynn et al. 2020). Finally, the classified de novo repeats were aligned against the corresponding genomes with repeatMasker.

## Supplementary Material

Supplementary material is available at *Genome Biology and Evolution* online.

## Supplementary Material

evae055_Supplementary_Data

## Data Availability

The raw data (HiFi, 10X, Hi-C and RNAseq) and the genome sequence are available at NCBI under the project PRJNA1022034. The genome sequence and the 2 annotations are also available publicly at https://bipaa.genouest.org/sp/coenonympha_arcania/.
