# Supplementary material for: Chromosome-Level Assembly and Annotation of the Pearly Heath Coenonympha arcania Butterfly Genome
Source: Genome Biol Evol. 2024 Mar 16;16(3):evae055. doi: 10.1093/gbe/evae055 (PMC10980516; doi:10.1093/gbe/evae055)

**Supplementary material**

**Chromosome-level assembly and annotation of the pearly heath butterfly *Coenonympha arcania genome***

Fabrice Legeai^1,2*^, Sandra Romain^1^, Thibaut Capblancq^3^, Paul Doniol-Valcroze^4^, Mathieu Joron^4^, Claire Lemaitre^1^, Laurence Després^3^

1 Univ Rennes, Inria, CNRS, IRISA, 35000, Rennes, France

2 IGEPP, INRAE, Institut Agro, Univ Rennes, 35653, Le Rheu, France

3 LECA, Université Grenoble Alpes, Université Savoie Mont Blanc, CNRS, Grenoble, France

4 CEFE, Université de Montpellier, CNRS, EPHE, IRD, Montpellier, France

*Author for Correspondence: Fabrice Legeai ([fabrice.legeai@inrae.fr](mailto:fabrice.legeai@inrae.fr))

**Supplementary Table 1:** Summary of the raw sequencing data produced and corresponding quality.

| Sample | Type | SRA ID | # Raw reads | raw data (bp) | Q20 (%) | Q30 (%) | GC% | % uniquely mapped reads |
| --- | --- | --- | --- | --- | --- | --- | --- | --- |
| ARC2 | Chromium 10X | SRX22724551 | 219289994 | 32893499100 | 93.32 | 85.35 | 39.93 | 94.87 |
| La Mure | Omni-C | SRX22702957 | 281775726 | 42266358900 | 93 | 84.95 | 44.31 | 99.58 |
| BST1 | Pacbio HiFi | SRX22685695 | 1413972 | 19742935004 | 98.86 | 97.32 | 38.61 | 100 |
| ArcC | RNASeq larva | SRX22724888 | 229531178 | 34429676700 | 96.76 | 91.47 | 45.82 | 67.97 |
| ArcA | RNASeq adult | SRX22724887 | 216296296 | 32444444400 | 96.73 | 91.27 | 42.56 | 72.19 |

**Supplementary Table 2: list of the Lepbase v4 proteomes (downloaded from http://download.lepbase.org/v4/sequence/) used for the Braker3 annotation.**

| Species | Version | # proteins |
| --- | --- | --- |
| Bicyclus anynana | BaGv2 | 29381 |
| ‍Calycopis cecrops | v1.1 | 16456 |
| ‍Danaus plexippus | v3 | 15130 |
| Heliconius erato demophoon | v1 | 20118 |
| Heliconius erato lativitta | v3 | 14613 |
| ‍Heliconius melpomene melpomene | Hmel2.5 | 21656 |
| ‍Junonia coenia | v2 | 17244 |
| ‍Lerema accius | v1.1 | 17411 |
| ‍Maniola jurtina | ilManJurt1.1 | 24115 |
| ‍Melitaea cinxia | v1 | 16668 |
| ‍Papilio glaucus | v1.1 | 15692 |
| ‍Papilio machaon | v1.0 | 15497 |
| ‍Papilio polytes | v1.0 | 15559 |
| ‍Papilio xuthus | v1.0 | 15322 |
| ‍Phoebis sennae | v1.1 | 16492 |
| ‍Pieris napi | v1.1 | 20325 |
| ‍Vanessa cardui | v1 | 14437 |

**Supplementary Table 3:** Annotation statistics of the BST1 genome

|  | **Braker3** | **Helixer** |
| --- | --- | --- |
| nb genes | 21557 | 21392 |
| nb transcripts | 26206 | 21392 |
| nb proteins | 26206 | 21392 |
| mean gene size | 10113.29 | 10233.93 |
| median gene size | 3596 | 5184 |
| mean transcript_size | 2709.80 | 2147.19 |
| median transcript size | 1907 | 1563.5 |
| ‍mean CDS size | 1461.22 | 1629.20 |
| ‍median CDS size | 1074 | 1083 |
| ‍mean 3’UTR size | 1167.87 | 385.46 |
| ‍median 3’UTR size | 401 | 133 |
| ‍mean 5’UTR size | 523.51 | 132.59 |
| ‍median 5’UTR size | 213 | 105 |
| Mean distance of the 3’UTR to the stop codon* | 1959.04 | 759.24 |
| ‍Median distance of the 3’UTR to the stop codon* | 441 | 223 |
| mean protein size | 487.07 | 543.07 |
| median protein size | 358 | 361 |
| percentage of BUSCO single | 89.92 | 94.00 |
| percentage of BUSCO duplicated | 1.25 | 1.70 |
| percentage of BUSCO fragmented | 0.91 | 1.44 |
| percentage of BUSCO missing | 7.92 | 2.86 |
| ‍percentage of assigned RNAseq reads** | 62.71 | 71.73 |
| percentage of unassigned RNAseq reads because of ambiguity (annotation overlaps)** | 10.32 | 0.50 |
| ‍Number of singletons (%)*** | 2702 (12.53) | 3589 (16.78) |
| ‍Number of genes in specific orthogroups (%)*** | 4160 (19.30) | 2133 (9.97) |
| complete NR proteins**** | 49158 | 42337 |
| complete WCP**** | 12 | 11 |
| complete OR**** | 16 | 18 |
| complete GR**** | 4 | 4 |
| complete CSP**** | 19 | 18 |
| complete OBP**** | 15 | 12 |
| complete IR**** | 11 | 12 |

* mean distance between the extreme position of the 3prime UTR and the stop codon
** among uniquely mapped reads (raw reads were trimmed, mapped with STAR aligner, PCR duplicates and multimapped reads were removed)
*** when comparing the 2 protein datasets with Orthofinder (in case of isoforms, the largest protein per gene was selected)
****matches with 60% identity over 80% of subject size

**Supplementary Table 4:**  Genome assembly statistics by chromosome.

|  | **chromosome** | **size** | **Hifi (ZW) median coverage** | **10x (ZZ) median coverage** | **Hifi polymorphism rate** | **repeat coverage** | GC percent |
| --- | --- | --- | --- | --- | --- | --- | --- |
| **scaffold_1** | Z | 25840627 | 20 | 34 | 0,0002 | 0,5958 | 37.34 |
| **scaffold_2** | 1 | 20680252 | 36 | 28 | 0,0132 | 0,4872 | 38.13 |
| **scaffold_3** | 2 | 20299349 | 37 | 30 | 0,0137 | 0,4625 | 38.20 |
| **scaffold_4** | 3 | 20283189 | 37 | 27 | 0,0119 | 0,5048 | 38.48 |
| **scaffold_5** | 4 | 20168739 | 37 | 29 | 0,0132 | 0,4770 | 38.22 |
| **scaffold_6** | 5 | 20009806 | 37 | 27 | 0,0131 | 0,4938 | 38.38 |
| **scaffold_7** | 6 | 19008510 | 37 | 29 | 0,0140 | 0,4855 | 37.91 |
| **scaffold_8** | 7 | 18995423 | 37 | 29 | 0,0134 | 0,4928 | 37.62 |
| **scaffold_9** | 8 | 18869206 | 36 | 26 | 0,0120 | 0,5135 | 38.76 |
| **scaffold_10** | 9 | 18757483 | 36 | 28 | 0,0121 | 0,5056 | 38.19 |
| **scaffold_11** | 10 | 18118210 | 36 | 27 | 0,0134 | 0,5061 | 37.96 |
| **scaffold_12** | 11 | 17935327 | 37 | 29 | 0,0136 | 0,4779 | 37.65 |
| **scaffold_13** | 12 | 17912571 | 36 | 25 | 0,0137 | 0,5134 | 38.65 |
| **scaffold_14** | 13 | 17436191 | 37 | 25 | 0,0126 | 0,5081 | 38.52 |
| **scaffold_15** | 14 | 17429161 | 37 | 27 | 0,0130 | 0,5033 | 38.22 |
| **scaffold_16** | 15 | 17176696 | 36 | 27 | 0,0147 | 0,4933 | 37.91 |
| **scaffold_17** | 16 | 16532000 | 36 | 26 | 0,0142 | 0,5006 | 37.94 |
| **scaffold_18** | 17 | 16500846 | 36 | 25 | 0,0137 | 0,5159 | 38.45 |
| **scaffold_19** | 18 | 16455962 | 36 | 24 | 0,0135 | 0,5050 | 38.53 |
| **scaffold_20** | 19 | 16050382 | 36 | 24 | 0,0131 | 0,5094 | 39.16 |
| **scaffold_21** | 20 | 15858292 | 36 | 25 | 0,0130 | 0,5280 | 38.81 |
| **scaffold_22** | 21 | 14114091 | 36 | 24 | 0,0163 | 0,5091 | 38.31 |
| **scaffold_23** | 22 | 13867112 | 33 | 7 | 0,0067 | 0,6229 | 40.81 |
| **scaffold_24** | 23 | 12215597 | 35 | 17 | 0,0123 | 0,5925 | 38.95 |
| **scaffold_25** | 24 | 11516504 | 36 | 22 | 0,0140 | 0,5231 | 39.09 |
| **scaffold_26** | 25 | 11251347 | 32 | 9 | 0,0087 | 0,6710 | 40.06 |
| **scaffold_27** | 26 | 10505419 | 33 | 16 | 0,0124 | 0,6208 | 38.78 |
| **scaffold_28** | 27 | 9202170 | 32 | 8 | 0,0084 | 0,6766 | 40.97 |
| **scaffold_29** | 28a | 8557086 | 34 | 16 | 0,0100 | 0,5790 | 39.82 |
| **scaffold_30** | 28b | 7852074 | 35 | 19 | 0,0115 | 0,5999 | 39.31 |
| **scaffold_31** | putative W | 3700072 | 33 | 0 | 0,0015 | 0,6511 | 40.01 |
| **scaffold_32** | putative W | 3257025 | 54 | 0 | 0,0015 | 0,6706 | 39.83 |
| **scaffold_33** | unplaced scaffold | 617018 | 31 | 0 | 0,0010 | 0,6599 | 42.17 |
| **scaffold_34** | unplaced scaffold | 149000 | 35 | 0 | 0,0013 | 0,7468 | 41.60 |
| **scaffold_35** | unplaced scaffold | 80190 | 57 | 7 | 0,0009 | 0,7094 | 37.43 |
| **scaffold_36** | unplaced scaffold | 52121 | 16 | 0 | 0,0004 | 0,9813 | 42.95 |
| ‍scaffold_37 | unplaced scaffold | 34954 | 0 | 0 | 0 | 0 | 64.16 |
| ‍scaffold_38 | unplaced scaffold | 30608 | 44 | 4 | 0 | 0.6292 | 37.36 |
| Mitochondrial genome‍ | Mitochondrial genome | 15313 | 251.5 | 15.5 | 0 | 0 | 20.13 |

**Supplementary Table 5:** Distribution of the orthogroups composition by species, (statistics with the Braker3 annotation are noted between parentheses)

| **species** | **class** | **site** | **version** | **# proteins** | **singletons** | **orthogroups** | **proteins in orthogroups** | **specific orthogroups** | **proteins in specific orthogroups** | **satyrinae orthogroups** | **proteins in satyrinae orthogroups** | **nymphalidae orthogroups** | **proteins in nymphalidae orthogroups** | **lepidoptera orthogroups** | **proteins in lepidoptera orthogroups** |
| --- | --- | --- | --- | --- | --- | --- | --- | --- | --- | --- | --- | --- | --- | --- | --- |
| *Coenonympha arcania* | [Nymphalidae](https://www.ncbi.nlm.nih.gov/Taxonomy/Browser/wwwtax.cgi?mode=Undef&id=33415&lvl=3&keep=1&srchmode=1&unlock) [Satyrinae](https://www.ncbi.nlm.nih.gov/Taxonomy/Browser/wwwtax.cgi?mode=Undef&id=42282&lvl=3&keep=1&srchmode=1&unlock) | BIPAA | OGS1 | 21392  (21557) | 1639  (848) | 12769  (11511) | 19753  (20709) | 337  (340) | 2163  (2415) | 229  (236) | 518  (781) | 1832  (1314) | 3107  (3740) | 10371  (9621) | 13965  (13773) |
| *Maniola jurtina* | Nymphalidae Satyrinae | Darwin Tree of Life | ilManJurt11 | 25734 | 278  (284) | 11609  (11637) | 25456  (25450) | 171  (180) | 860  (931) | 274  (296) | 760  (796) | 737  (740) | 1523  (1561) | 10427  (10421) | 22313  (22162) |
| *Pararge aegeria* | Nymphalidae Satyrinae | Darwin Tree of Life | ilParAegt1.1 | 20956 | 189  (202) | 11393  (11414) | 20767  (20754) | 104  (96) | 476  (415) | 226  (257) | 730  (847) | 695  (703) | 1304  (1314) | 10368  (10358) | 18257  (18178) |
| *Heliconius melpomene* | Nymphalidae Heliconiinae | Lepbase | v2.5 | 21656 | 2076  (2126) | 12767  (12748) | 19580  (19530) | 245  (260) | 1257  (1511) | 0 | 0 | 2065  (2030) | 3723  (3514) | 10457  (10458) | 14600  (14505) |
| *Danaus plexippus* | Nymphalidae Danainae | Lepbase | v4 | 15130 | 1111  (1165) | 12057  (12012) | 14019  (13965) | 43  (47) | 117  (123) | 0 | 0 | 1664  (1623) | 1933  (1893) | 10350  (10342) | 11969  (11949) |
| *Ithomia salapia* | [Nymphalidae](https://www.ncbi.nlm.nih.gov/Taxonomy/Browser/wwwtax.cgi?mode=Undef&id=33415&lvl=3&lin=f&keep=1&srchmode=1&unlock) [Danainae](https://www.ncbi.nlm.nih.gov/Taxonomy/Browser/wwwtax.cgi?mode=Undef&id=127218&lvl=3&lin=f&keep=1&srchmode=1&unlock) | BIPAA | OGS1.0 | 42489 | 5260  (5452) | 15439  (15496) | 37229  (37037) | 1878  (1926) | 8461  (9287) | 0 | 0 | 2960  (2970) | 9848  (9020) | 10601  (10600) | 18920  (18730) |
| *Melinaea marsaeus* | Nymphalidae Danainae | BIPAA | OGS1.0 | 55449 | 10687  (11175) | 14804  (14778) | 44762  (44274) | 2228  (2273) | 14656  (14817) | 0 | 0 | 2889  (2822) | 15766  (12578) | 9687  (9683) | 14340  (16879) |
| *Bombyx mori* | Heteroneura | NCBI | Bmori_2016v1.0 | 27309 | 485  (498) | 11437  (11463) | 26824  (26811) | 399  (427) | 2003  (2149) | 0 | 0 | 0 | 0 | 11038  (11036) | 24821  (24662) |

**Supplementary Figure 1: (**A) Dorsal and ventral vues of the BST1 female specimen used for HiFi sequencing and (B) Blob plot of the genome. The X axis indicates the GC content of each scaffold, and the Y axis indicates the coverage with Hifi reads. In blue the scaffolds corresponding to Arthropoda.


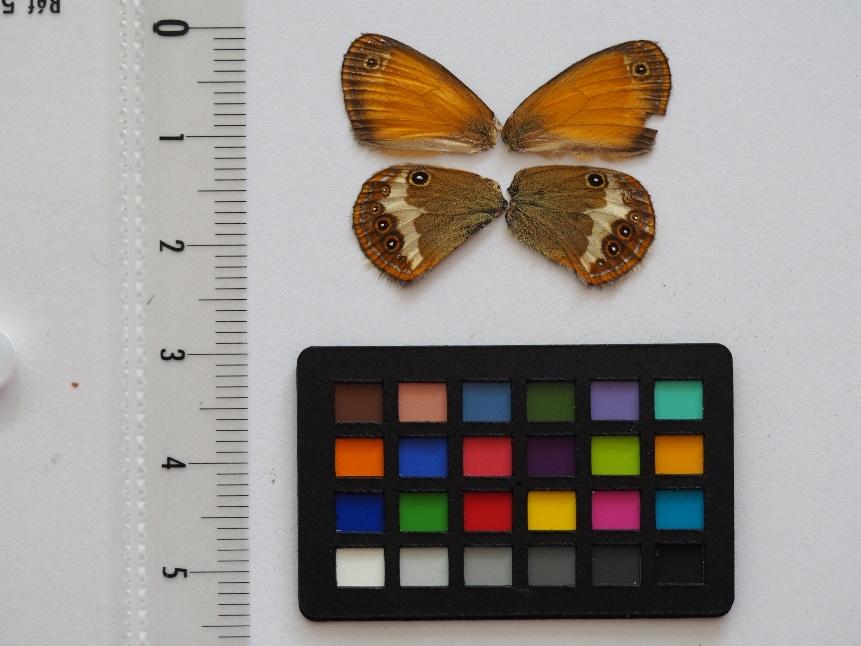
A


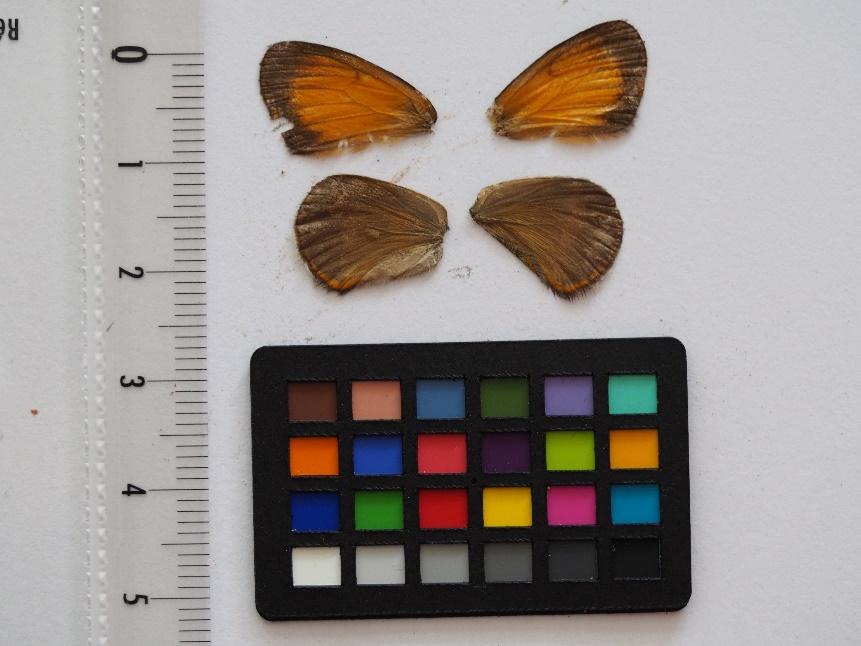


B
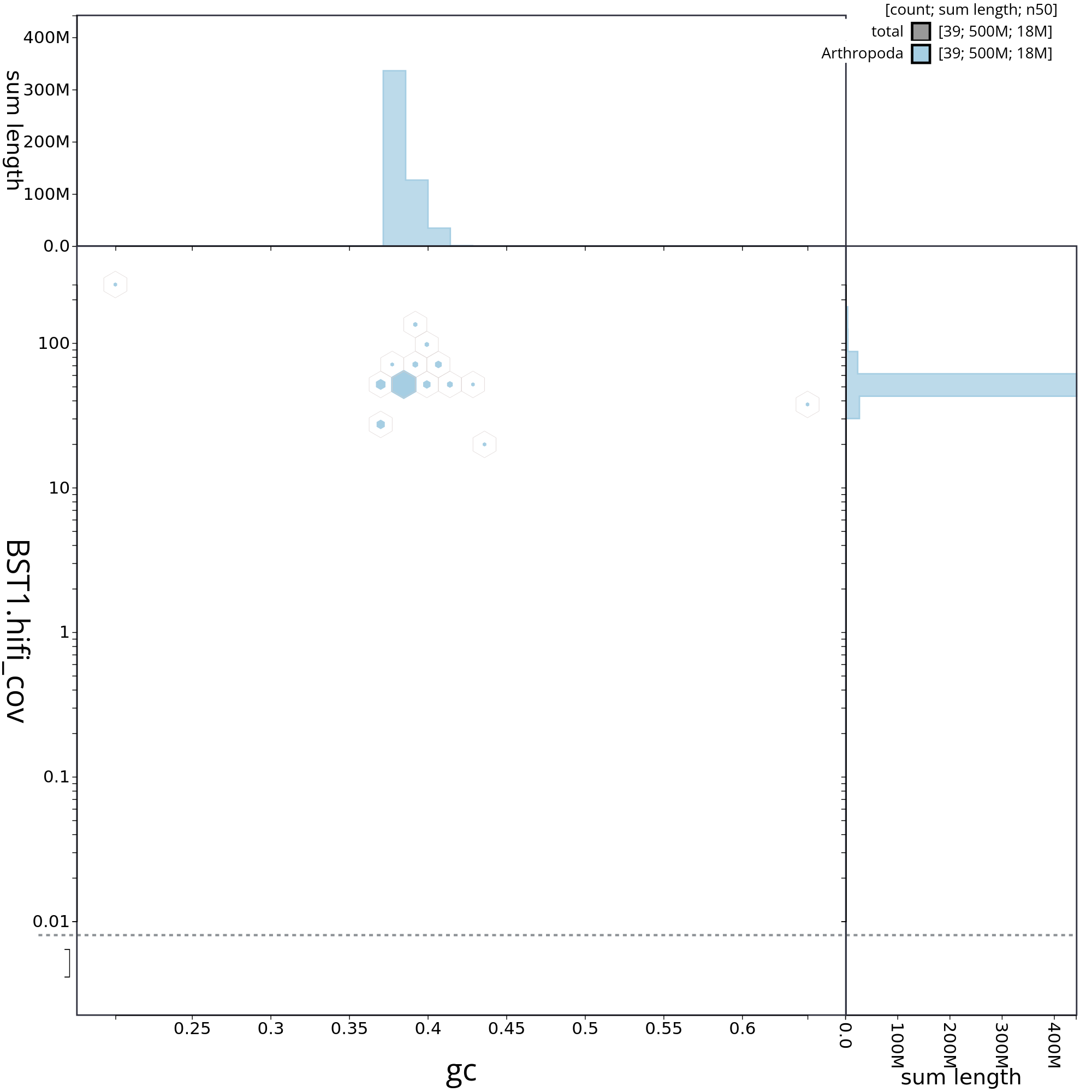


**Supplementary Figure 2:** Comparison of kmer abundances between raw HiFi reads and the genome assembly of *C. arcania*. The main curve represents the histogram of 31-mer abundances in the raw read set, with the y-value being the number of distinct 31-mers whose abundance in the read set is exactly the X value. Each vertical bar is colored by the number of times each kmer is present in the sequence assembly (haploid). The histogram shows two modes, as expected for a highly heterozygous organism, with the first one representing kmers in heterozygous loci and the second one homozygous kmers. We can see that the genome assembly contains very few spurious sequence duplications due to heterozygosity as most of the homozygous kmers are present exactly once in the assembly, and roughly half of the hetereozygous kmers are absent in the haploid assembly.


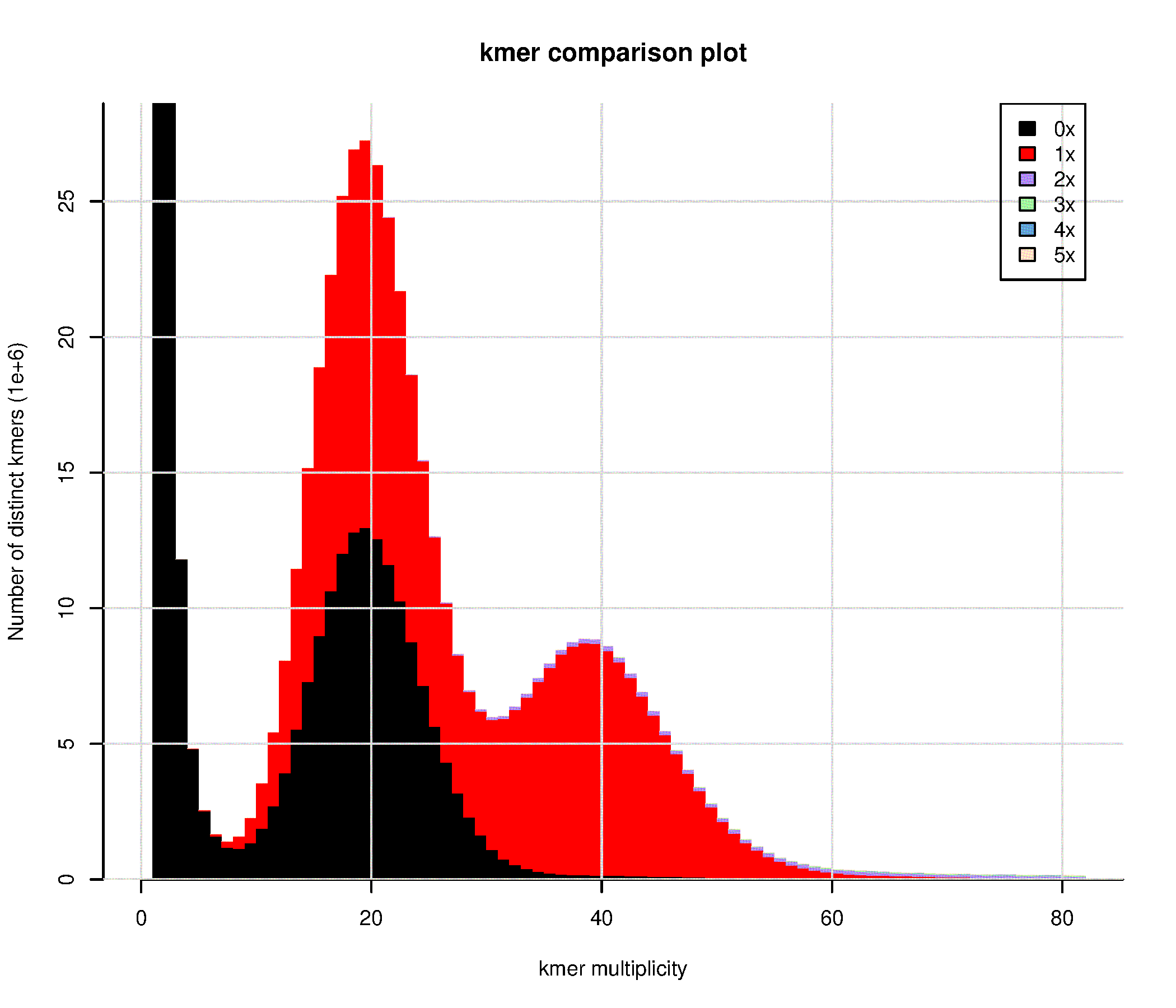


**Supplementary figure 3:**  Length distribution of 3' and 5' untranslated regions (UTRs) for transcripts obtained from both annotation procedures (Helixer and Braker3+GUSHR). Additionally, UTR sequences from three other Nymphalidae species *Heliconius melpomene*, *Danaus plexippus*, and *Melitae cinxia* were retrieved from UTRdb 2.0 (respectively Hmel1.54. Dplex_v4.54 and MelCinx1.0.54*)* for comparative analysis. The term "length" in this context refers to the cumulative size of the exon part corresponding to a non-translated region within each transcript.


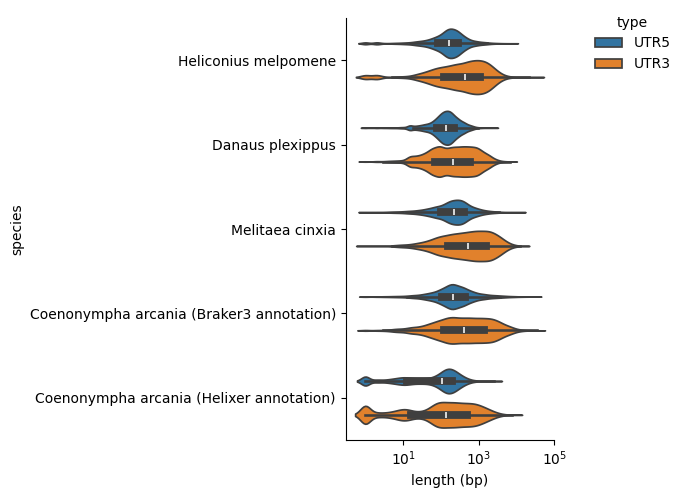


**Supplementary Figure 4:** Phylogenetic tree of the species used for creating orthogroups (BMOR =*Bombyx mori*, MMAR = *Melinaea marsaeus*, ISAL = *Ithomia salapia*, DPLE = *Danaus plexippus*, HMEL = *Heliconius melpomene*, PAEG = *Pararge aegeria*, MJUR = *Maliona jurtina,* CARC = *Coenonympha arcania*) and number of genes for each species that are singletons, duplicated species-specific, specific to Satyrinae, Nymphalidae or Lepidoptera.


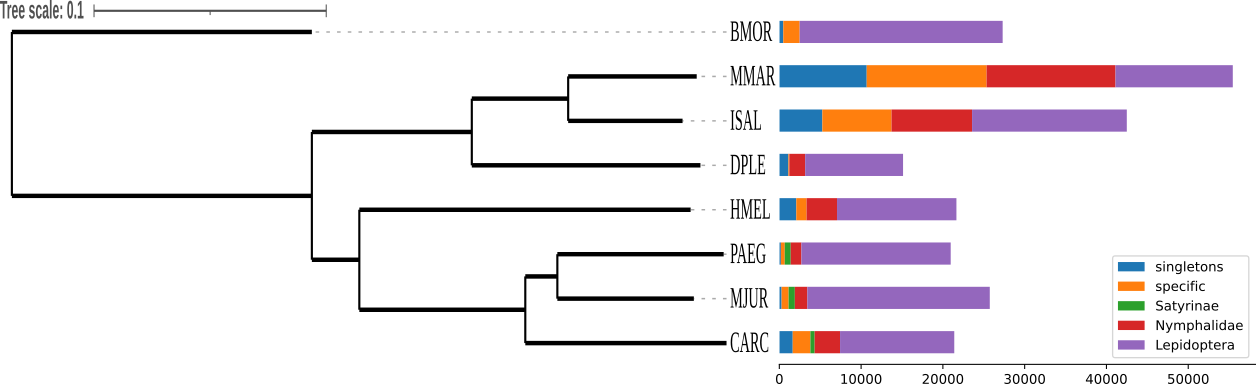


**Supplementary Figure 5: (**A) Repeat content of the 3 Satyrinae genomes calculated by similarity to known repeats (RepeatMasker with Insecta database). (B) Repeat content of the 3 Satyrinae genomes calculated by a *de novo* approach (RepeatModeler and classifier). Retrotransposons are reported in red. Numbers indicate the percentage of the genome covered by each repeat type. (C) Circos plot of the synteny blocks between *Coenonympha arcania* and *Maniola jurtina* obtained by aligning whole genome sequences. Each line drawn represents a syntenic block of at least 500 bp between one *C. arcania* (Carc) chromosome (grey box) and one *M. jurtina* (Mjur) chromosome (colored box). Synteny lines are colored after the *M. jurtina* chromosome they are linked to. We observe an overall high level of whole genome synteny between *C. arcania* and *M. jurtina*, with the exception of the W chromosome of *M. jurtina* for which the identified synteny blocks seem spuriously distributed on various *C. arcania* assembly chromosomes.


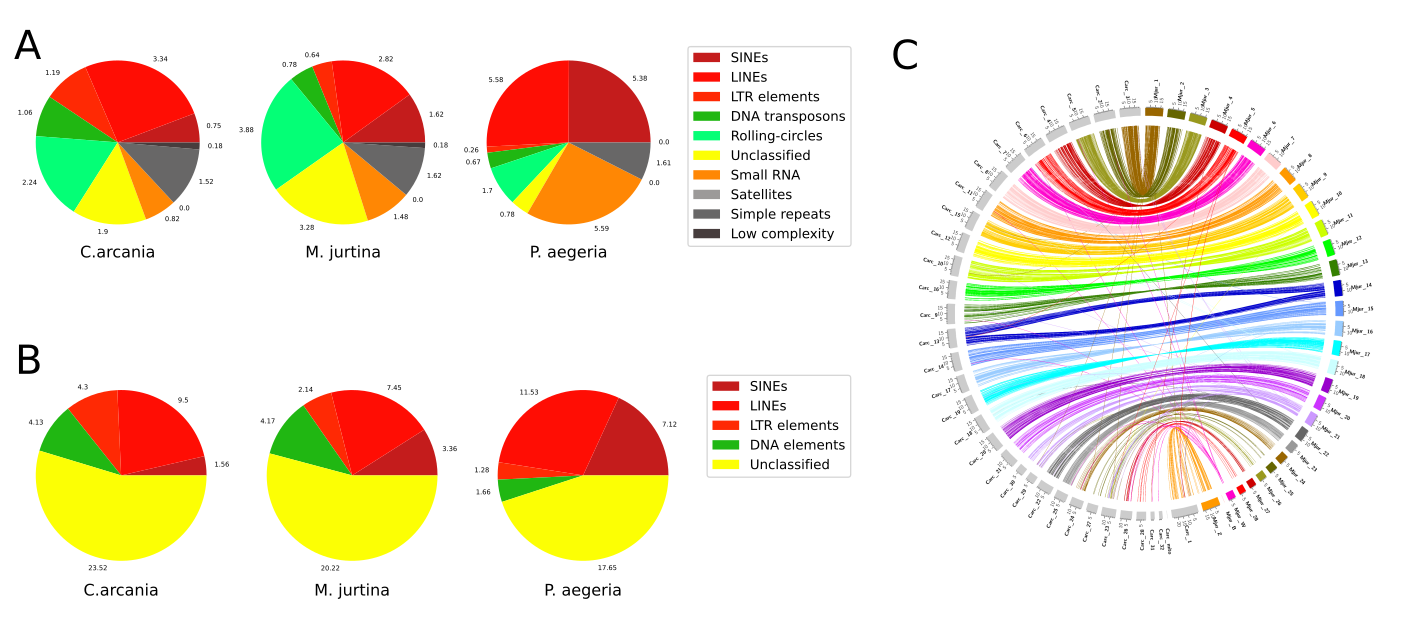

Supplement: evae055_Supplementary_Data [file evae055_supplementary_data.docx]
